# Associations of epigenetic age acceleration at birth and age 12 years with adolescent cardiometabolic risk: the HOME study

**DOI:** 10.1186/s13148-024-01779-8

**Published:** 2024-11-19

**Authors:** Jennifer L. Arzu, Karl T. Kelsey, George D. Papandonatos, Kim M. Cecil, Aimin Chen, Scott M. Langevin, Bruce P. Lanphear, Kimberly Yolton, Jessie P. Buckley, Joseph M. Braun

**Affiliations:** 1 Department of Epidemiology, School of Public Health, Brown University, 121 South Main Street, Providence, RI 02903 USA; 2 Department of Pathology and Laboratory Medicine, Brown University, Providence, RI USA; 3 Department of Biostatistics, School of Public Health, Brown University, Providence, RI USA; 4 Department of Environmental and Public Health Sciences, University of Cincinnati College of Medicine, Cincinnati, OH USA; 5 Department of Pediatrics, University of Cincinnati College of Medicine, Cincinnati, OH USA; 6 Department of Radiology, University of Cincinnati College of Medicine, Cincinnati, OH USA; 7 Department of Biostatistics, Epidemiology and Informatics, University of Pennsylvania Perelman School of Medicine, Philadelphia, PA USA; 8 Larner College of Medicine, University of Vermont, Burlington, VT USA; 9 University of Vermont Cancer Center, Burlington, VT USA; 10 Faculty of Health Sciences, Simon Fraser University, Burnaby, BC V5A 1S6 Canada; 11 Department of Pediatrics, Cincinnati Children’s Hospital Medical Center, University of Cincinnati College of Medicine, Cincinnati, OH USA; 12 Department of Epidemiology, University of North Carolina Gillings School of Global Public Health, Chapel Hill, NC USA

**Keywords:** Epigenetic age, Cardiometabolic risk, Adolescence, Developmental origins of health and disease, Epidemiology

## Abstract

**Background:**

Cardiometabolic risk factors among youth are rising. Epigenetic age acceleration, a biomarker for aging and disease-risk, has been associated with adiposity in children, but its association with other cardiometabolic risk markers remains understudied. We employed data from the Health Outcomes and Measures of the Environment (HOME) study, a prospective pregnancy and birth cohort in the greater Cincinnati metropolitan area, to examine whether accelerated epigenetic age at birth as well as accelerated epigenetic age and faster pace of biological aging at age 12 years were associated with higher cardiometabolic risk in adolescents.

**Results:**

After adjusting for potential confounders, including estimated cell type proportions, epigenetic gestational age acceleration at birth, derived from the Bohlin, Knight, and Haftorn clocks using cord blood DNA methylation data, was not associated with cardiometabolic risk z-scores or individual cardiometabolic risk score components (visceral fat, leptin to adiponectin ratio, HOMA-IR, triglycerides to HDL-C ratio, HbA1c, or systolic blood pressure) at age 12 years. We also did not observe any associations of epigenetic age acceleration, calculated with Horvath’s skin and blood, Hannum’s, and Wu’s epigenetic clocks using peripheral blood at age 12 years, with these same cardiometabolic risk markers. In contrast, faster pace of biological aging was associated with higher cardiometabolic risk [βs (95% CIs)] cardiometabolic risk score 0.25 (0.07, 0.42); visceral fat 0.21 (0.05, 0.38); and hemoglobin A1c 0.23 (0.05, 0.41) per standard deviation increase in pace of biological aging. Faster pace of biological aging was also positively associated with systolic blood pressure, triglycerides to HDL-C ratio, HOMA-IR, and leptin to adiponectin ratio, although these associations were not statistically significant.

**Conclusions:**

Our findings provide evidence that faster pace of biological aging was associated with higher cardiometabolic risk score, visceral fat, and HbA1c at age 12 years. Further research is needed to determine whether these associations persist from adolescence through adulthood.

**Supplementary Information:**

The online version contains supplementary material available at 10.1186/s13148-024-01779-8.

## Background

The prevalence of factors associated with elevated cardiometabolic risk among youth is rising [1–3]. Among United States (U.S.) youth ages 12–19 years, it is estimated that nearly 25% of have elevated blood glucose, 18% elevated waist circumference, 20% low high-density lipoprotein cholesterol (HDL-C), 5% elevated triglycerides, and 4% metabolic syndrome [4]. These risk factors track from childhood to adulthood and contribute to adult cardiovascular disease, the leading cause of global deaths, thus underscoring the critical need for preventive measures and intervention strategies in early life [5, 6].

Several social, behavioral, and environmental factors have been associated with cardiometabolic risk across the lifespan [6]. A potential biological mechanism underlying these associations is epigenetic aging. Epigenetic aging is a biomarker of biological age increasingly used to predict age-related disease risk [7]. It is estimated from the DNA methylation at select CpG sites across the genome using epigenetic clocks [7]. Several epigenetic clocks, many which use different CpG sites, have been developed to measure epigenetic gestational age (EGA) in neonates and quantify epigenetic age (EA) in children and adults [8–13]. These epigenetic clocks were constructed using different DNA methylation profile microarrays and training populations, as well as various tissue types [8–14]. Moreover, different generations of the clocks have emerged. First-generation clocks were derived from CpG sites correlated with chronological age and are used to calculate epigenetic age acceleration (EAA; residual obtained from regressing chronological age on estimated epigenetic age) [8–13], while a recently developed third-generation clock, DunedinPACE, quantifies the pace of biological aging and was trained on 19 longitudinal biomarkers linked to the pulmonary, cardiovascular, metabolic, renal, hepatic, periodontal, and immune systems [15]. Multiple epigenetic clocks have been associated with several disease risk factors (e.g., smoking and low socioeconomic status) and adverse health outcomes (e.g., cardiovascular disease, cancer, and mortality), but these associations vary with each clock [16–19].

Among children and adolescents, results from prior studies examining the associations of EAA and cardiometabolic risk factors have been inconsistent and the associations of pace of biological aging with cardiometabolic risk have been scarce [20–25]. While two studies found that epigenetic gestational age acceleration (EGAA) estimated via the Bohlin clock was associated with lower likelihood of rapid weight gain during childhood [20, 21] and lower weight at 10 years of age [21], another study reported that EAA at birth, estimated via the Horvath clock, was associated with greater fat mass and faster gain in weight and BMI during childhood and adolescence [22]. However, this same study found that EAA at age 7 years was not associated with adiposity during childhood and adolescence [22]. In late adolescence, Hannum EAA at age 17 years was associated with greater lean mass, BMI, and waist circumference, while Horvath EAA was associated with higher leptin levels and HOMA-IR [23]. For blood pressure, both positive and inverse associations with EGAA at birth and EAA in childhood and adolescence have been reported, although the 95% confidence intervals included the null [23, 25]. Only one study to date has examined the associations of EAA with lipid biomarkers among children, with findings indicating that EAA at age 17 years was both inversely and positively associated, although not significantly, with total cholesterol, triglycerides, HDL-C, and low-density lipoprotein cholesterol (LDL-C) in late adolescence [23]. Further, the associations of pace of biological aging with cardiometabolic risk factors in adolescence are unknown.

Therefore, using six cardiometabolic risk biomarkers assessed at age 12 years, we examined whether accelerated EGA at birth as well as accelerated EA and faster pace of biological aging at age 12 years were associated with higher cardiometabolic risk in adolescence. Because of the variability in the design of the epigenetic clocks, we also assessed the deviations and correlations between the estimated epigenetic age and the observed age (gestational or chronological) across three widely used EGA clocks and three EA clocks.

## Methods

### Study design and participants

We used data from the Health Outcomes and Measures of the Environment (HOME) Study, a prospective pregnancy and birth cohort in the greater Cincinnati metropolitan area [26, 27]. Between March 2003 and January 2006, the HOME study enrolled 468 out of 1263 eligible pregnant women from nine prenatal practices affiliated with three hospitals in the Cincinnati, Ohio region [26]. Pregnant women were eligible for inclusion if they: 1) were at least age 18 years; 2) were 16 ± 3 weeks of gestation; 3) were fluent in English; 4) resided in the study region in a home built prior to 1978 (i.e., when the U.S federal government banned consumer used of lead-based paint) with goal of including children with increased lead exposure given that one of the aims of the HOME study was to test the efficacy of lead hazard controls on children’s lead exposure and health) [28]; 5) not residing in a mobile or trailer home; 6) expected to continue prenatal care and deliver at a collaborating hospital; 7) were HIV negative; 8) were not taking seizure, thyroid, or chemotherapy/radiation medications; 9) had no diagnosis of diabetes, bipolar disorder, schizophrenia, or cancer requiring radiation or chemotherapy [26].

Of the 468 enrolled women, 412 women and their children were eligible for the 12-year visit after 67 women dropped out prior to delivery, but 11 of them re-enrolled at a later follow-up (Supplemental Fig. 1) [27]. Women commonly dropped out because of time commitment and family member’s unwillingness to participate [26]. Among 242 singleton children who completed the 12-years follow-up visit [27], 181 singleton children had epigenetic gestational age at birth, at least one cardiometabolic risk biomarker at age 12 years, and complete covariate data. A total of 183 singleton children had epigenetic age at age 12 years, at least one cardiometabolic risk biomarker, and complete data on relevant covariates.

HOME study protocols were approved by the Institutional Review Boards (IRBs) at the Cincinnati Children’s Hospital Medical Center (CCHMC) and collaborating delivery hospitals [26]. The Centers for Disease Control and Prevention and Brown University deferred to the CCHMC IRB as the IRB of record. Women provided written informed consent for themselves and their children [26]. At the 12-year visit, adolescents provided written informed assent [27].

### DNA methylation

We collected venous blood samples at delivery (cord blood) and age 12 years, and stored them at − 80 °C prior to DNA methylation (DNAm) analysis as previously described [29]. Briefly, trained laboratory technicians quantified DNAm at > 850,000 CpG sites per sample using the Illumina EPIC Bead Chip (EPIC) v1.0 array [30]. Quality control and normalization of the DNAm data were performed using the *minfi* Bioconductor package in R (v1.48.0) [29, 31, 32]. We excluded samples with more than 5% high detection *p*-values (*p*-value > 1 × 10^–6^) and filtered out probes with at least one high detection *p*-value (*p*-value > 1 × 10^–6^), known to be cross-reactive, having single nucleotide polymorphisms, or mapping to sex chromosomes [29, 33]. A total of 669,622 probes were used for further analysis. We used normal-exponential convolution using out-of-band probes (Noob) for background and dye bias correction [34], Beta MIxture Quantile (BMIQ) normalization for probe design bias correction [35], and ComBat for batch effect adjustment [36].

### Epigenetic age

We used the preprocessed DNAm to estimate: 1) epigenetic gestational age (EGA) at birth using three cord-blood-based EGA clocks—Bohlin, Knight, and Haftorn [8–10]; 2) epigenetic age (EA) at age 12 years using the Horvath skin and blood, Hannum, and Wu clocks [11–13]; and 3) pace of biological aging with the recently developed DunedinPACE clock [15]. We have selected these epigenetic clocks because they are frequently used in other studies, allowing us to compare our results. Moreover, these selected clocks were trained in tissue types (i.e., cord blood for EGA clocks and blood for EA clocks) consistent with the specimens collected in our cohort [8–13, 15]. Further, because we quantified DNA methylation using the Illumina EPIC array, we included at least one clock in each period (i.e., birth and age 12 years) and generation (i.e., first and third generation) trained using DNA methylation from the EPIC array. Blood-based pediatric-specific epigenetic clocks are limited [14], and as a result we examined epigenetic clocks at age 12 years even if the training population did not cover this age range. We also considered Levine’s PhenoAge, a second-generation epigenetic clock trained on chronological age and 9 clinical biomarkers, but excluded it due to a high median absolute error (medAE = 25.3 years) in our cohort [37].

Details of each epigenetic clock [8–13, 15] and comparison of their characteristics have been previously reported [14, 16]. Briefly, the first-generation epigenetic clocks—Bohlin, Knight, Haftorn, Horvath, Hannum, and Wu—are predictors of age and were developed by regressing gestational or chronological age on DNAm at CpG sites primarily via elastic net regression [8–13]. In contrast, DunedinPACE, a third-generation clock and predictor of the pace/rate of aging, was based on 19 biomarkers repeatedly measured from young to middle adulthood [15]. Across the epigenetic clocks, variability in the design, demographics of populations used for training the data, and selected CpG sites resulted in little to no overlap in CpGs between the clocks (Supplemental Fig. 2) [8–13, 15]. Therefore, we used multiple epigenetic clocks to assess consistency in the effect estimates from the regression analyses.

Epigenetic age (EGA and EA) and their respective acceleration measures (EGAA and EAA) were computed using the *methylclock* R package (v1.8.0) [38]. EGAA and EAA were estimated from the residuals obtained after regressing EGA on gestational age and EA on chronological age (*methylclock v1.8.0*) [38]. Positive values of EGAA and EAA indicate accelerated epigenetic aging, epigenetic age that is greater than chronological age. Due to the known associations of age acceleration with cell type proportions [39], we calculated intrinsic measures of EGAA and EAA by adjusting for estimated cell type proportions. We used the reference-based approach developed by Gervin et al. [40] for cord blood and by Salas et al. [41] for adult peripheral blood, as implemented by *methylclock* to estimate cell type proportions [38]. Pace of biological aging, defined as an average rate of change in one year of biological aging per year of chronological aging [15], was estimated using the DunedinPACE algorithm and the intrinsic measure was separately calculated in a linear regression model adjusted for estimated cell type proportions; neutrophils were excluded due to multicollinearity [41]. Missing CpG probes required for estimating each first-generation clock were recovered using the k-nearest neighbors (*k* = 10) imputation approach in *methylclock* consistent with imputation method used by the Knight, Wu, and Hannum epigenetic clocks [9, 12, 13, 38], while missing probes for DunedinPACE were rescued using the mean imputation procedure in the *PACEProjector* function of the DunedinPACE algorithm (v0.99.0) [15].

### Adolescent cardiometabolic risk

We measured several cardiometabolic risk biomarkers at the 12-years visit [27]. Trained laboratory technicians at the Cincinnati Children’s Hospital Medical Center Clinical Translational Research Center Core Laboratory quantified serum insulin, glucose, HDL-C, triglycerides, adiponectin, leptin, and hemoglobin A1C (HbA1c) concentrations using immunoassays conducted on fasting blood samples. We obtained three sitting blood pressure measurements at 1-min intervals using a Dinamap Pro100 automated monitor and used the average of the second and third measures for analyses. Cross-sectional area of fat inside the abdominal cavity (cm^2^) was determined by dual-energy X-ray absorptiometry (DXA) using a Horizon densitometer (Hologic) [27]. We estimated the ratios of triglycerides to HDL-C and leptin to adiponectin. The homeostatic model assessment for insulin resistance (HOMA-IR) was calculated as: fasting insulin (mIU/L) × fasting glucose (mg/dL)/405 [42].

We derived a summary cardiometabolic risk score as previously described [43]. In brief, we calculated age and sex standardized z-scores for HOMA-IR, HbA1c, triglycerides to HDL-C ratio, leptin to adiponectin ratio, and cross-sectional area of fat inside the abdominal cavity (visceral fat) from the studentized residuals of the linear regression for each component [43, 44]. Prior to standardization, we log_2_-transformed HOMA-IR, triglycerides to HDL-C ratio, leptin to adiponectin ratio, and visceral fat, because they were right-skewed. Systolic blood pressure was first nationally standardized according to sex, age, and height measures, and subsequently standardized internally to ensure comparability with the individual components of the cardiometabolic risk score [45, 46]. The summed standardized z-scores for HOMA-IR, triglycerides to HDL-C ratio, systolic blood pressure, leptin to adiponectin ratio, and visceral fat comprised the cardiometabolic risk score, with a higher score indicative of higher cardiometabolic risk [43].

### Covariates

We used previous literature and directed acyclic graphs (DAG) to identify potential confounders associated with epigenetic age at birth or age at 12 years and early adolescent cardiometabolic risk (Supplemental Figs. 3 and 4). Trained research staff abstracted data from medical charts and used standardized computer-assisted interviews to obtain information on maternal age, maternal education, household income, gestational age at delivery, birthweight, as well as the child’s age, sex, and race/ethnicity [26, 27]. We calculated pre-pregnancy body mass index (BMI) from self-reported height and weight [47]. For mothers with missing self-reported weight, we imputed their weight with super learning [47, 48]. With the *SuperLearner* (v2.0–22) R package, we used a tenfold cross-validation approach to obtain weighted predictions based on a subset of candidate algorithms [47]. Prenatal tobacco smoke exposure was assessed using the average log_10_-transformed serum cotinine concentrations [49]. Serum cotinine concentrations were measured using high-performance liquid chromatography-tandem mass spectroscopy from blood samples collected from mothers via venipuncture at 16 or 26 weeks of gestation [49]. To obtain estimated cell type proportions, we used the reference-based algorithm developed by Gervin et al. [40] for cord blood and by Salas et al. [41] for adult peripheral blood. At the 12-year study visit, adolescents used images illustrating the Tanner stages of development to self-assess their pubertal status based on pubic hair growth [27, 50].

### Statistical analyses

Using descriptive statistics, we summarized the distribution (median and interquartile range for continuous variables, frequency and proportion for categorical variables) of sociodemographic and perinatal characteristics of the mother and children in the study sample. We tested differences in characteristics for included and excluded participants using Pearson’s chi-squared or Fisher’s exact test for categorical variables, and Wilcoxon rank sum test for continuous measures. We reported the mean and standard deviation, overall and by covariates, of EGAA for the Bohlin, Knight, and Haftorn epigenetic clocks; EAA for the Wu, Hannum, and Horvath’s skin and blood epigenetic clocks; and pace of biological aging for DunedinPACE.

To evaluate the performance of the first-generation epigenetic clocks, we calculated Spearman correlations and median absolute error (medAE) between epigenetic age and age at birth or 12 years for each first-generation epigenetic clock.^15^ The median absolute error was calculated as the median absolute difference between epigenetic age and chronological age. We additionally used Spearman rank correlations to investigate the relationship between EGAA at birth and EAA at age 12 years.

To estimate the association of intrinsic EGAA at birth and EAA at age 12 years with cardiometabolic risk, we fit separate multivariable linear regression models with EGAA or EAA, respectively, as predictors and with cardiometabolic risk score, each of the individual cardiometabolic risk components (i.e., HOMA-IR, triglycerides to HDL-C ratio, systolic blood pressure, leptin to adiponectin ratio, and visceral fat), and HbA1c at age 12 years as outcomes. We standardized EGAA, EAA, and pace of biological aging using z-scores (i.e., mean and SD were set to 0 and 1, respectively) to ensure comparability of effect sizes across the different epigenetic clocks. We adjusted all models for pre-pregnancy BMI (continuous), gestational serum cotinine (< 0.015 ng/mL as unexposed; 0.015–3 ng/mL as secondhand exposure; ≥ 3 ng/mL as active smoking), maternal age at delivery (continuous, years), household income (continuous, USD/year), child age at outcome measurement (continuous, years), child sex (male; female), and child race/ethnicity (non-Hispanic, white; non-Hispanic, black; other). Estimated cell type proportions were accounted in the intrinsic accelerated epigenetic age measures of all first-generation clocks; and thus, were not adjusted for in the linear regression models [40, 41]. For pace of biological aging, we adjusted for estimated cell type proportions in the linear regression models. We excluded stage of pubertal development because it was a potential intermediate in the relationship between EAA and cardiometabolic risk in our DAG (Supplemental Fig. 4) [51, 52].

To test the robustness of our results, we conducted two sensitivity analyses: 1) we additionally adjusted models for birthweight in the analyses examining the associations of cardiometabolic risk with intrinsic EAA and pace of biological aging but excluded it in the associations of EGAA with cardiometabolic risk because it is a potential intermediate [22, 53]; and 2) we evaluated the associations of cardiometabolic risk with EGAA, EAA, and pace of biological aging without adjusting for cell type proportions.

Results were considered statistically significant when the two-sided *p*-value < 0.05. We did not adjust for multiplicity because we aimed to explore associations of epigenetic age measures with cardiometabolic risk biomarkers. We conducted statistical analyses using R (version 4.3.2) [54].

## Results

### Characteristics of study participants

The median gestational age at birth among the 181 singleton children included in our analysis was 39.4 weeks (IQR = 38.7–40.0), with 55% being female and 60% non-Hispanic white (Supplemental Table 1; Table 1). Nearly two-thirds of mothers had an annual household income > $40,000 during pregnancy (62%), and 60% had gestational serum cotinine concentrations indicative of secondhand tobacco smoke exposure (0.015 – 3 ng/mL, 60%). At the 12-year visit, the median age of children included in the analysis was 12.3 years (IQR = 11.9–12.8). While no significant differences in characteristics were detected between the included and excluded participants at birth and the 12-years visit, a higher proportion of children included in this analysis were non-Hispanic white with mothers who were > 25–35 years old at delivery (Supplemental Table 1).

**Table 1:** Epigenetic gestational age acceleration (EGAA) at birth across covariates among children from the HOME study (2003–2006), *N* = 181

Covariates		Mean EGAA at birth (SD)		
		ageAccelBohlin^a^	ageAccelKnight^a^	ageAccelHaftorn^a^
Overall		− 0.12 (0.63)	− 0.32 (1.47)	− 0.16 (0.82)
Child sex				
Female	100 (55.2%)	− 0.13 (0.63)	− 0.15 (1.52)	− 0.18 (0.83)
Male	81 (44.8%)	− 0.11 (0.62)	− 0.54 (1.38)	− 0.14 (0.80)
Child race				
Non-hispanic white	109 (60.2%)	− 0.04 (0.62)	− 0.16 (1.56)	− 0.20 (0.85)
Non-hispanic black	62 (34.3%)	− 0.30 (0.60)	− 0.59 (1.29)	− 0.13 (0.75)
Other^b^	10 (5.5%)	− 0.03 (0.68)	− 0.44 (1.33)	− 0.05 (0.94)
Maternal age at delivery (years)				
18–25	40 (22.1%)	− 0.22 (0.67)	− 0.27 (1.45)	− 0.18 (0.84)
> 25–35	116 (64.1%)	− 0.10 (0.63)	− 0.31 (1.53)	− 0.14 (0.83)
> 35	25 (13.8%)	− 0.09 (0.51)	− 0.47 (1.21)	− 0.27 (0.76)
Annual household income				
< $20,000	38 (21%)	− 0.32 (0.65)	− 0.65 (1.54)	− 0.19 (0.82)
$20,000–40,000	30 (16.6%)	− 0.13 (0.61)	− 0.03 (1.35)	0 (0.70)
$40,000–80,000	63 (34.8%)	− 0.13 (0.62)	− 0.46 (1.35)	− 0.28 (0.85)
> $80,000	50 (27.6%)	0.04 (0.60)	− 0.08 (1.59)	− 0.09 (0.84)
Pre-Pregnancy BMI (kg/m^2^)				
< 25	76 (42%)	− 0.05 (0.65)	− 0.13 (1.56)	− 0.12 (0.87)
≥ 25–30	59 (32.6%)	− 0.10 (0.65)	− 0.37 (1.49)	− 0.17 (0.86)
≥ 30	46 (25.4%)	− 0.28 (0.52)	− 0.58 (1.26)	− 0.24 (0.66)
Gestational serum cotinine (ng/mL)				
< 0.015 (unexposed)^c^	55 (30.4%)	0.06 (0.63)	− 0.05 (1.64)	0.02 (0.88)
0.015–3 (secondhand)	109 (60.2%)	− 0.18 (0.62)	− 0.37 (1.39)	− 0.26 (0.79)
> 3 (active smoking)	17 (9.4%)	− 0.36 (0.55)	− 0.93 (1.25)	− 0.13 (0.71)

SD, Standard Deviation; BMI, body mass index

^a^Extrinsic epigenetic measures are reported (i.e., not adjusted for cell type proportions).

^b^Other category includes children of American Indian, Asian/Pacific, Hispanic, and Unknown race/ethnicity.

^c^Below detection limit.

### Differences in EGAA and EAA across sociodemographic characteristics

The children in our sample were on average decelerated at birth (Table 1i.e., lower EGAA than expected), and had average EAA (i.e., EAA near zero) and slower pace of biological aging than expected at age 12 years (Table 2). Across all clocks, there was variability in mean EGAA and EAA across levels of some participants’ characteristics. For instance, male children had higher acceleration than females based on the Hannum clock, while female children had higher acceleration based on skinHorvath clock. In contrast, children who were non-Hispanic black and whose mothers had a pre-pregnancy BMI ≥ 30 had the lowest mean EGAA at birth based on all three EGA clocks (Table 1). Children with mothers > 35 years of age at delivery and with pre-pregnancy BMI ≥ 30 had the lowest mean EAA on the Wu, Hannum, and skinHorvath clocks (Table 2). Mean pace of biological aging was higher with lower annual household income and higher gestational serum cotinine concentrations (Table 2).

**Table 2:** Epigenetic age acceleration (EAA) and pace of biological aging at age 12 years across covariates among children from the HOME study (2003–2006), *N* = 183

Covariates		Mean EAA at age 12 years (SD)			DunedinPACE^a^
		ageAccelWu^a^	ageAccel Hannum^a^	ageAccel skinHorvath^a^	
Overall		− 0.01 (1.09)	0.02 (3.90)	0.05 (3.59)	0.87 (0.08)
Child sex					
Female	98 (53.6%)	− 0.01 (1.08)	− 0.11 (4.15)	0.16 (3.71)	0.86 (0.09)
Male	85 (46.4%)	0 (1.11)	0.17 (3.61)	− 0.07 (3.47)	0.88 (0.08)
Child race					
Non-hispanic white	100 (54.6%)	0.10 (1.19)	0.17 (4.18)	0.06 (3.83)	0.84 (0.07)
Non-hispanic black	71 (38.8%)	− 0.19 (0.98)	− 0.24 (3.71)	0.09 (3.46)	0.90 (0.09)
Other^b^	12 (6.6%)	0.16 (0.76)	0.32 (2.48)	− 0.18 (2.27)	0.90 (0.07)
Maternal age at delivery (years)					
18–25	48 (26.2%)	0.06 (0.96)	0.07 (3.79)	0.59 (3.78)	0.87 (0.10)
> 25–35	109 (59.6%)	0.01 (1.18)	0.09 (4.08)	− 0.01 (3.68)	0.86 (0.08)
> 35	26 (14.2%)	− 0.22 (0.92)	− 0.36 (3.41)	− 0.69 (2.7)	0.87 (0.08)
Annual household income					
< $20,000	46 (25.1%)	− 0.16 (0.98)	− 0.10 (3.38)	0.10 (3.54)	0.90 (0.10)
$20,000–40,000	30 (16.4%)	0.13 (1.20)	− 0.22 (4.31)	0.44 (4.59)	0.86 (0.08)
$40,000–80,000	59 (32.2%)	− 0.06 (1.06)	0.05 (4.07)	− 0.24 (3.01)	0.86 (0.07)
> $80,000	48 (26.2%)	0.12 (1.17)	0.25 (3.99)	0.13 (3.69)	0.84 (0.07)
Pre-pregnancy BMI (kg/m^2^)					
< 25	76 (41.5%)	0.11 (1.04)	0.07 (3.73)	0.16 (3.66)	0.85 (0.08)
≥ 25–30	62 (33.9%)	− 0.02 (1.27)	0.26 (4.38)	0.24 (3.98)	0.86 (0.08)
≥ 30	45 (24.6%)	− 0.18 (0.90)	− 0.40 (3.53)	− 0.37 (2.88)	0.89 (0.09)
Gestational serum cotinine (ng/mL)					
< 0.015 (unexposed)^c^	52 (28.4%)	− 0.06 (1.05)	− 0.25 (3.95)	− 0.39 (3.14)	0.85 (0.07)
0.015–3 (secondhand)	112 (61.2%)	0.03 (1.11)	0.07 (3.91)	0.15 (3.59)	0.86 (0.09)
> 3 (active smoking)	19 (10.4%)	− 0.05 (1.16)	0.48 (3.87)	0.69 (4.71)	0.91 (0.10)

SD, Standard Deviation; BMI, body mass index

^a^Extrinsic epigenetic measures are reported (i.e., not adjusted for cell type proportions).

^b^Other category includes children of American Indian, Asian/Pacific, Hispanic, and Unknown race/ethnicity.

^c^Below detection limit.

### Differences between the epigenetic clocks in the correlations and deviations of epigenetic age and observed age

**Fig. 1:**
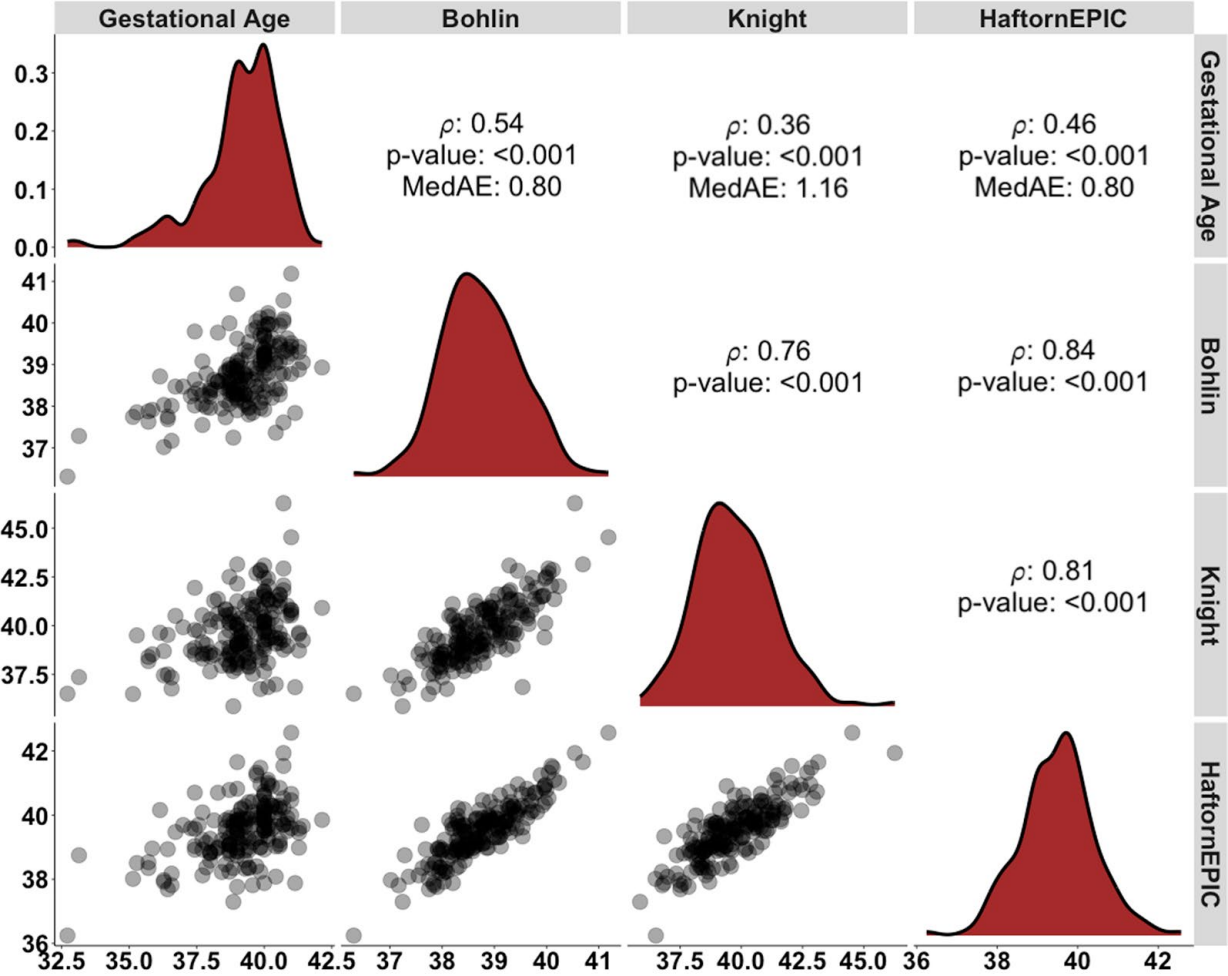
Spearman rank correlations of gestational age and epigenetic gestational age at birth (weeks) among singleton children from the HOME study (*N* = 181)

**Fig. 2:**
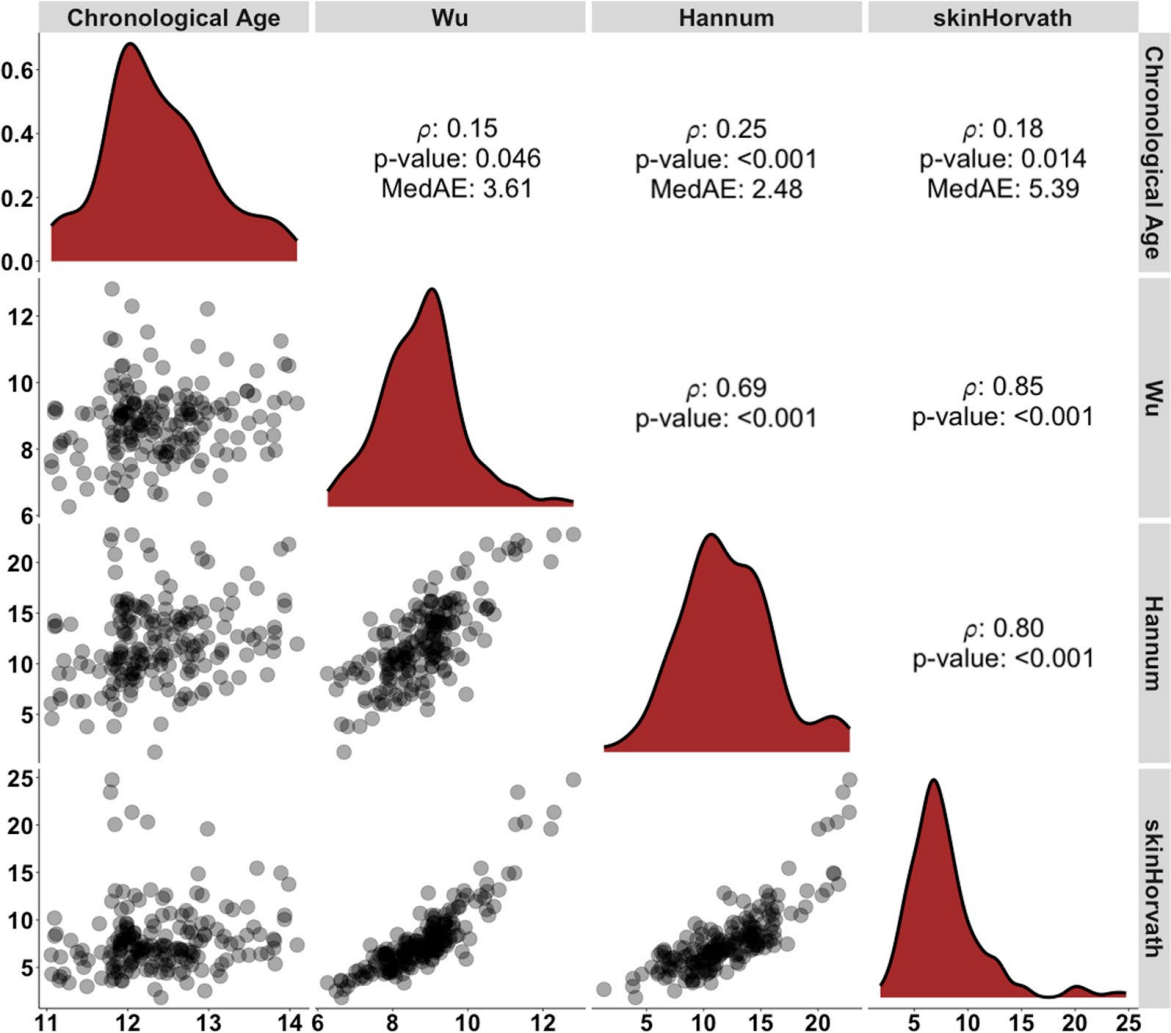
Spearman rank correlations of chronological age and epigenetic age (years) at age 12 years among singleton children from the HOME study (*N* = 183)

Gestational age at delivery (weeks) was more strongly correlated with the Bohlin EGA clock (*ρ* = 0.54) than the Haftorn EGA (*ρ* = 0.46, *p* < 0.001) and Knight clock (*ρ* = 0.36, *p* < 0.001) (Fig. 1). The median absolute error for the EGA clocks ranged from 0.80 to 1.22 weeks, with Bohlin and Haftorn having the lowest medAE and Knight having the highest medAE. Although the correlations of chronological age (years) with EA at age 12 years were weak (ρ range: 0.15–0.25), they were consistently positive across all EA clocks (Fig. 2). Among the EA clocks at age 12 years, Hannum EA had the lowest median absolute error (medAE = 2.48 years), whereas skinHorvath EA had the highest (medAE = 5.39 years).

### Correlations of EGAA at birth with EAA and pace of biological aging at age 12 years

**Fig. 3:**
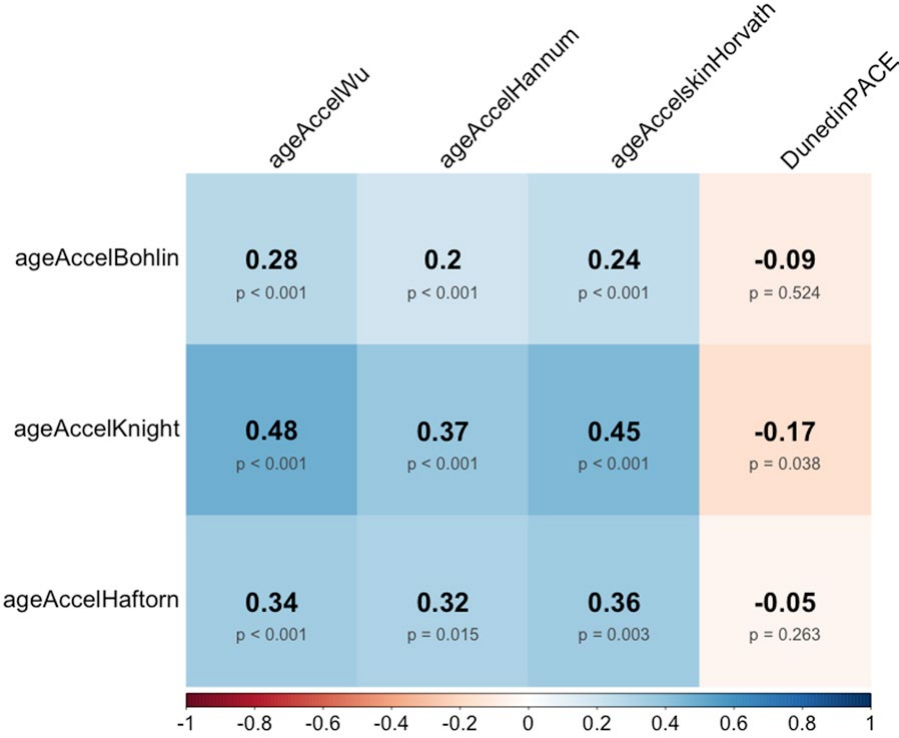
Spearman rank correlations of epigenetic gestational age acceleration (EGAA) at birth with epigenetic age acceleration (EAA) and pace of biological aging at age 12 years, among singleton children from the HOME study with DNA methylation data at birth and age 12 years (N = 152)

Among the 152 singleton children with DNA methylation data at birth and age 12 years, we observed consistent positive correlations between EGAA at birth and EAA at age 12 years (Fig. 3). EGAA at birth was inversely correlated with pace of biological aging (Bohlin *ρ* = − 0.09, *p* = 0.524; Knight *ρ* = − 0.17, *p* = 0.038; Haftorn *ρ* = − 0.05, *p* = 0.263), although the correlations of pace of biological aging with Bohlin EGAA and Haftorn EGAA were not statistically significant.

### Adjusted associations of EGAA at birth and EAA/pace of biological aging at age 12 years with cardiometabolic risk in adolescence

**Fig. 4:**
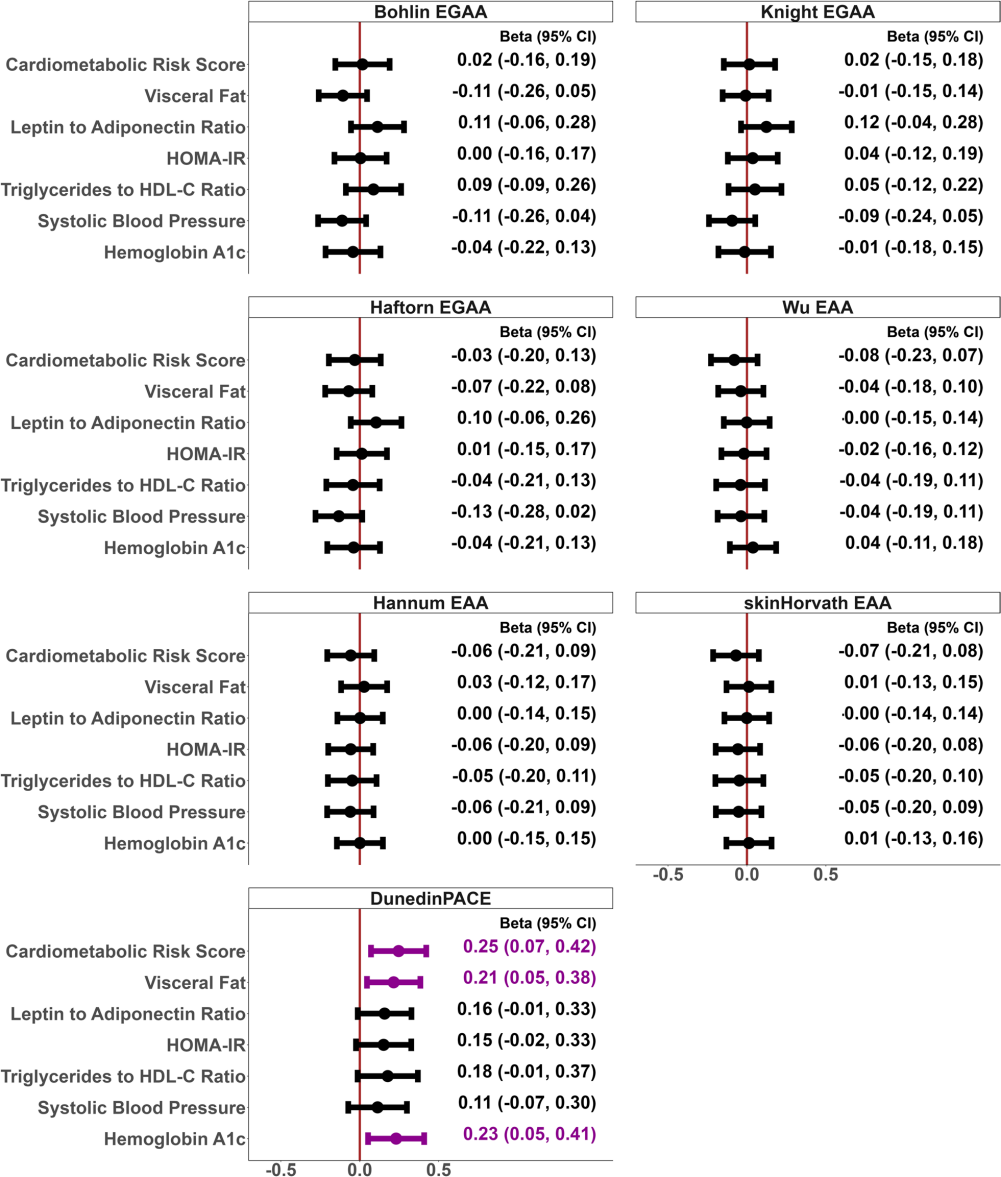
Adjusted associations of intrinsic epigenetic gestational age acceleration (EGAA) at birth, and epigenetic age acceleration (EAA) and pace of biological aging at 12 years with adolescent cardiometabolic risk

In covariate-adjusted analysis, EGAA at birth had consistent positive associations with leptin to adiponectin ratio but inverse associations with visceral fat and systolic blood pressure across the 3 EGA clocks, although the 95% confidence intervals included the null (Fig. 4). Wu, Hannum, and Horvath EAA at age 12 years were consistently associated with lower cardiometabolic risk score, HOMA-IR, triglycerides to HDL-C ratio, and systolic blood pressure, but the 95% confidence intervals included the null. We did not observe any other consistent patterns of associations of EGAA at birth with cardiometabolic risk score, triglycerides to HDL-C ratio, and HOMA-IR; and of EAA at age 12 years with visceral fat and HbA1c.

Faster pace of biological aging was associated with higher cardiometabolic risk score, leptin to adiponectin ratio, visceral fat, HOMA-IR, HbA1c, triglycerides to HDL-C ratio, and systolic blood pressure at age 12 years (Fig. 4). After adjusting for covariates, as the pace of biological aging increased by one SD (0.08), the cardiometabolic risk score, visceral fat, and HbA1c increased, on average, by 0.25 SD (95% CI 0.07–0.42), 0.21 SD (95% CI 0.05–0.38), and 0.23 (95% CI 0.05–0.41), respectively. Further adjustment for birthweight in sensitivity analyses yielded similar results (Supplemental Fig. 5). When excluding the estimated cell type proportions in sensitivity analyses, we observed that the associations of pace of biological aging with cardiometabolic risk score, visceral fat, leptin to adiponectin ratio, and HOMA-IR were strengthened while the associations with triglycerides to HDL-C ratio, HbA1c, and systolic blood pressure were attenuated (Supplemental Fig. 6).

Each circle represents the effect estimate of each association and the error bars represent the corresponding 95% confidence intervals. Associations with *p* < 0.05 are displayed in purple.

Adjusted standardized difference in cardiometabolic risk score or individual cardiometabolic risk component at age 12 years per 1 SD increase in EGAA at birth or EAA/pace of biological aging at age 12 years. CI = Confidence Interval; Intrinsic = adjusted for estimated cell type proportions. Adjusted for pre-pregnancy BMI (continuous, kg/m^2^), gestational serum cotinine (< 0.015 ng/mL as unexposed; 0.015–3 ng/mL as secondhand exposure; ≥ 3 ng/mL as active smoking), maternal age at delivery (continuous, years), household income (continuous, USD/year), child age (continuous, years), child sex (male; female), child race/ethnicity (non-Hispanic White; non-Hispanic Black; other), and estimated cell type proportions (at birth: nucleated red blood cells, CD8^+^ T-cells, CD4^+^ T-cells, natural killer cells, B-cells, monocytes, and granulocytes; at 12-year visit: eosinophils, basophils, monocytes, naïve and memory B cells, naïve and memory CD4^+^ and CD8^+^ T cells, natural killer, and T regulatory cells; neutrophils were excluded due to multicollinearity).

## Discussion

Our results indicate that faster pace of biological aging, as measured by DunedinPACE, was associated with elevated overall cardiometabolic risk score, as well as higher visceral fat and hemoglobin A1c. Adjusting for estimated cell type proportions, given that epigenetic clocks are known to vary with the immune cell composition [39], strengthened the associations of pace of biological aging with some cardiometabolic risk biomarkers including triglycerides to HDL-C ratio, HbA1c, and systolic blood pressure, but attenuated the associations with others, such as cardiometabolic risk score and visceral fat. Currently, only one study has investigated the relationship of pace of biological aging with biomarkers of cardiometabolic risk in adolescents [55]. In the Child Health Study, a cohort of 439 children ages 8–13 years with and without history of maltreatment based on the Pennsylvania’s Statewide Child Welfare Information System, higher BMI z-score was associated with faster Dunedin methylation pace of biological aging (DunedinPoA) [55]. However, the analysis assessed the influence of BMI on pace of biological aging, and not vice versa.

While we found no evidence of significant associations between accelerated epigenetic measures and early adolescent cardiometabolic risk markers, our data was compatible with EGAA at birth being positively associated with leptin to adiponectin ratio but inversely associated with systolic blood pressure. Among 1115 mother–offspring pairs of the Generation R Study, Bohlin EGAA at birth was also inversely associated with systolic blood pressure at age 10 years, although the results included the null [25]. The positive association of skinHorvath EAA with systolic blood pressure at age 10 years in the Generation R Study as well as Hannum EEAA and Horvath IEAA with systolic blood pressure at age 17 years in the Raine Study contrasted our data, which was most compatible with an inverse association of EAA with systolic blood pressure at age 12 years [23, 25]. Further, in two studies examining the relationship of age acceleration at birth with adiposity in children, Bohlin EGAA at birth was associated with elevated weight from birth to 9 months but lower weight at age 10 years, which was consistent with our findings of Bohlin EGAA with visceral fat at age 12 years [20, 21], but differed from findings of another study reporting that Horvath EAA at birth was associated with higher fat mass, and rapid gain in weight and BMI during childhood and adolescence [22].

Compared with the consistently positive associations of the cardiometabolic risk markers with pace of biological aging—calculated from third-generation clock DunedinPACE, the associations of EGAA and EAA, derived from first-generation clocks, with the cardiometabolic risk biomarkers reflected both positive and inverse associations. Differences in these associations may be due to how the first-generation clocks were trained compared to the third-generation clock DunedinPACE. DunedinPACE was trained on 19 clinical biomarkers with the goal of quantifying the rate of aging and capturing decline in organ system function [15]. Thus, it may be more sensitive at assessing disease risk. In contrast, the first-generation clocks were exclusively trained on gestational or chronological age, where accelerated epigenetic aging measures represent deviations between epigenetic and chronological age [8–13, 15].

We evaluated the deviations and correlations of estimated epigenetic age and observed age across six first-generation epigenetic clocks, three EGA clock at birth, and three EA clocks during early adolescence in our study due to variability in their design and prior studies showing that estimates may vary based on the clock [16, 20, 56–61]. In our study, the Bohlin clock had the strongest correlation and lowest median absolute error between gestational age and EGA at birth. These findings align with prior studies indicating that the Bohlin clock more accurately estimates EGA at birth compared with the Knight clock [20, 56–58] and Haftorn clock [59]. However, in some cohorts, the Bohlin and Haftorn clocks performed similarly [60, 61]. At age 12 years, we observed low correlations between chronological age and EA, with the Hannum clock displaying the strongest correlation. Although the Wu and skinHorvath clocks included children in their training populations, the broad age ranges spanning multiple stages of childhood—and adulthood for skinHorvath—may have affected the precision of their estimates and influenced their performance in our cohort [11, 14]. Taken together, differences in performance across the clocks could stem from the variability in how the epigenetic clocks were developed, including the demographics and sample size of the training population and selected CpGs, and results for them should be interpreted in the context of these differences [8–13].

Our findings suggest that pace of biological aging could be used as a biomarker to monitor cardiometabolic health as early as childhood and adolescence. Faster rate of biological aging has implications for increased risk of disease (e.g., cardiovascular disease) and mortality [15, 16]. Accelerated epigenetic aging in adolescence has been associated with higher risk of cardiovascular disease in middle adulthood, which may be consequential to premature mortality [23]. Therefore, identifying individuals aging faster could inform early intervention strategies and prevent the onset of adverse health outcomes in adulthood.

Our study has some strengths including the assessment of age acceleration during two critical windows of development in childhood using multiple epigenetic clocks at birth and at age 12 years as well as the inclusion of various cardiometabolic risk biomarkers in our analysis. However, this study has some limitations. First, the cross-sectional nature of the associations at the 12-years visit precludes us from determining their directionality and extrapolating causality. Prior cross-sectional analyses among children have found inverse associations of cardiometabolic risk factors with epigenetic age accelerations and pace of biological aging [55, 62]. Our findings should be validated in longitudinal analyses examining the pace of biological aging with changes in cardiometabolic risk through childhood and adolescence. Second, we tested the associations of multiple epigenetic-based measures with several cardiometabolic risk outcomes, which may have resulted in findings due to random chance. Therefore, we have focused on the patterns of associations. Third, estimates of epigenetic age using adult-specific clocks such as the Hannum clock may have reduced precision given that they were trained exclusively on adult populations. However, in our cohort, the Hannum epigenetic clock had stronger correlation and lower median absolute error than the pediatric-specific Wu epigenetic clock. Fourth, we assessed cardiometabolic risk using a composite score with equal weights allocated to each individual cardiometabolic risk component. Notably, findings were generally consistent across the individual components of the cardiometabolic risk score.

Further, there is potential for selection bias due to the exclusion of adolescents with missing data or from loss to follow-up. Sociodemographic characteristics of participants who returned for follow-up visits were comparable to those of the full cohort [27]. In addition, not all eligible pregnant women enrolled into the study, which may have induced self-selection bias. Reporting bias may have been introduced with self-reported data such as maternal weight and height. There may be residual confounding by factors such as race/ethnicity given the heterogeneity of individuals within racial categories. Finally, given our study eligibility criteria, our cohort may not be representative of births in the study region; thus, our findings may not be generalizable to other populations [27].

## Conclusions

Our results suggest that faster pace of biological aging may influence cardiometabolic risk, visceral fat, and hemoglobin A1c during early adolescence. However, we did not find evidence that accelerated EGA at birth or EA at age 12 years were associated with early adolescent cardiometabolic risk. Future research should further explore this relationship using repeated measures of DunedinPACE and cardiometabolic risk markers at different stages during adolescence and adulthood.

## Supplementary Information

Additional file1.

## Data Availability

Data are available upon request. The HOME study Principal Investigators welcome new collaborations with other investigators and actively engage in collaborative data-sharing projects. Interested investigators should visit https://homestudy.research.cchmc.org/contact or contact JMB (joseph_braun_1@brown.edu) and KY (kimberly.yolton@cchmc.org) to obtain additional information about The HOME study, discuss collaborative opportunities, and request a project proposal form. The HOME study Protocol Review Committee reviews proposed research projects to ensure that they do not overlap with extant projects and are an efficient use of scarce resources (e.g., biospecimens).
