# Supplementary material for: Associations of epigenetic age acceleration at birth and age 12 years with adolescent cardiometabolic risk: the HOME study
Source: Clin Epigenetics. 2024 Nov 19;16:163. doi: 10.1186/s13148-024-01779-8 (PMC11577890; doi:10.1186/s13148-024-01779-8)
Supplement: Supplementary file 1 — Additional file1. [file 13148_2024_1779_MOESM1_ESM.docx]

**Supplemental Material For**: Associations of Epigenetic Age Acceleration at Birth and Age 12 Years with Adolescent Cardiometabolic Risk: The HOME Study

Authors: Jennifer L. Arzu, Karl T. Kelsey, George D. Papandonatos, Kim M. Cecil, Aimin Chen, Scott M. Langevin, Bruce P. Lanphear, Kimberly Yolton, Jessie P. Buckley, Joseph M. Braun

Table of Contents

[Supplemental Figure 1. Flow chart of study participants in the HOME Study by available data on DNA methylation, cardiometabolic risk biomarkers, and covariates. 2](#_Toc181606456)

[Supplemental Figure 2. Non-overlapping and overlapping CpG probes across pediatric and adult epigenetic age (EA) clocks used in the analyses. 3](#_Toc181606457)

[Supplemental Figure 3. Directed acyclic graphs for the relationship of epigenetic gestational age acceleration (EGAA) at birth with adolescent cardiometabolic risk. 4](#_Toc181606458)

[Supplemental Figure 4. Directed acyclic graphs for the relationship of epigenetic age acceleration (EAA) at age 12 years with adolescent cardiometabolic risk. 5](#_Toc181606459)

[Supplemental Figure 5. Forest plots of the adjusted associations of intrinsic epigenetic age acceleration (EAA) and pace of biological aging at age 12 years with adolescent cardiometabolic risk, adjusted for birthweight (sensitivity analyses). 7](#_Toc181606460)

[Supplemental Figure 6. Forest plots of the adjusted associations of extrinsic epigenetic gestational age acceleration (EGAA) at birth, and epigenetic age acceleration (EAA) and pace of biological aging at age 12 years with adolescent cardiometabolic risk (sensitivity analyses): No adjustment for estimated cell type proportions. 8](#_Toc181606461)


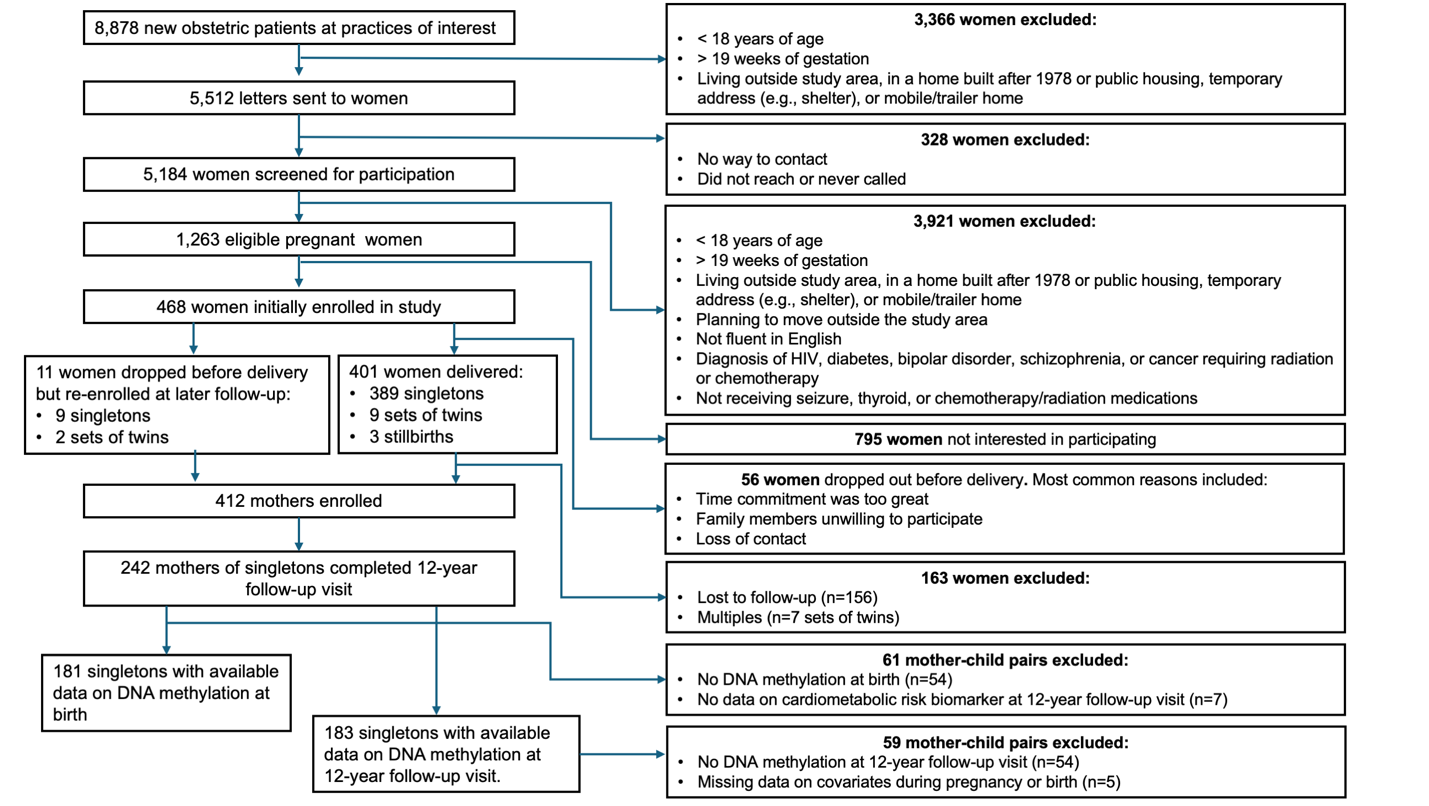


# Supplemental Figure 1. Flow chart of study participants in the HOME Study by available data on DNA methylation, cardiometabolic risk biomarkers, and covariates.


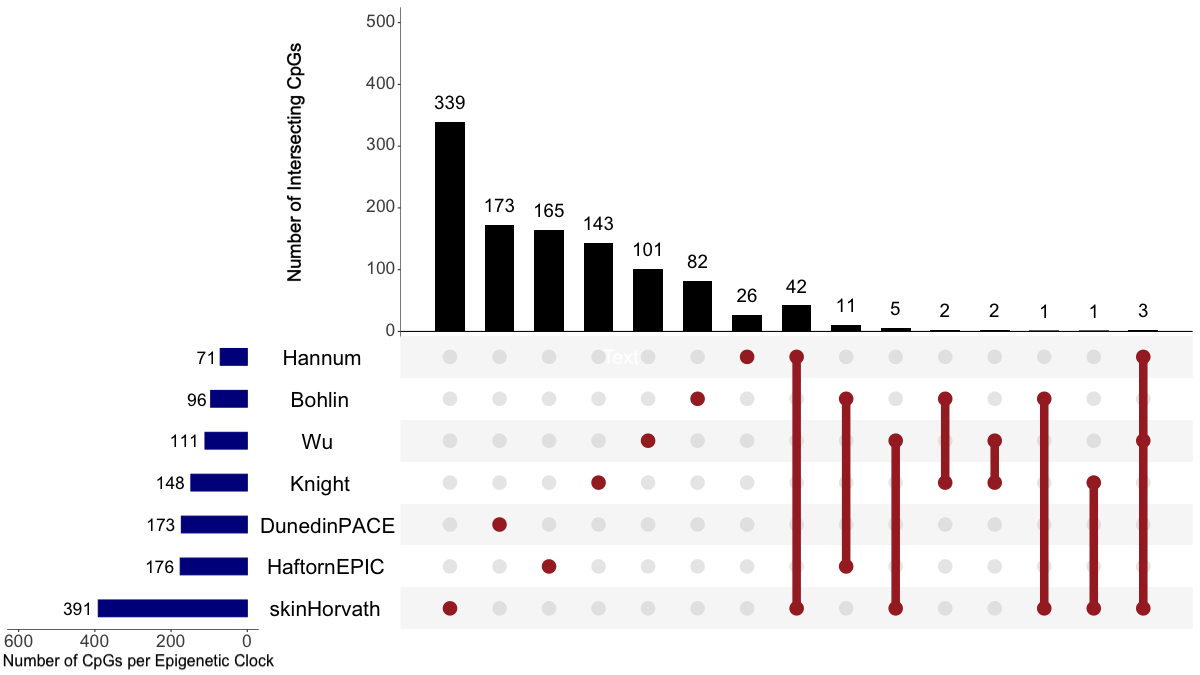


# Supplemental Figure 2. Non-overlapping and overlapping CpG probes across pediatric and adult epigenetic age (EA) clocks used in the analyses.

Single red dots represent non-overlapping CpGs for each epigenetic clock. Connected dots correspond to CpGs overlapping between epigenetic clocks. Black bars display the number of non-overlapping or overlapping CpGs for the epigenetic clocks.


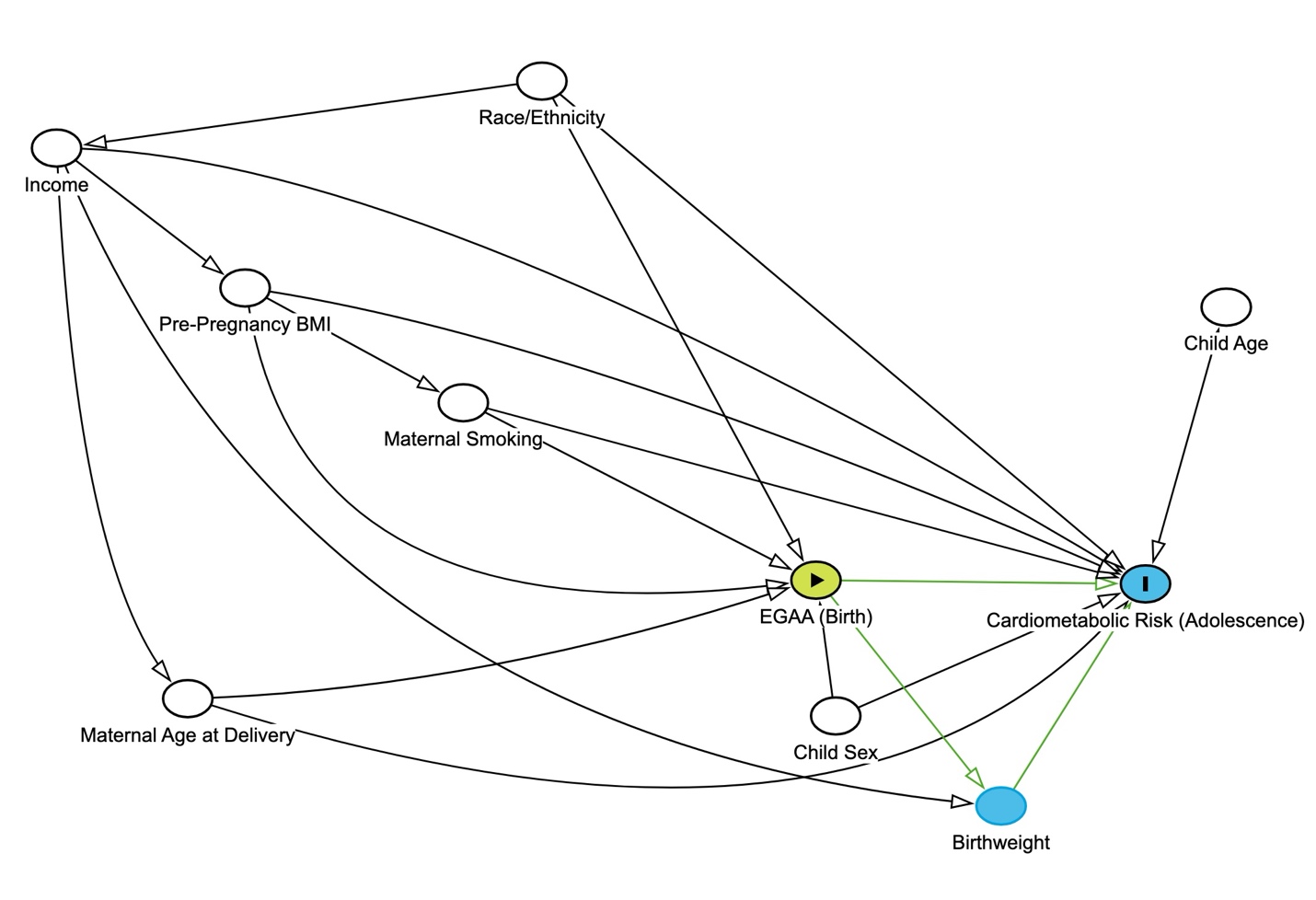


# Supplemental Figure 3. Directed acyclic graphs for the relationship of epigenetic gestational age acceleration (EGAA) at birth with adolescent cardiometabolic risk.


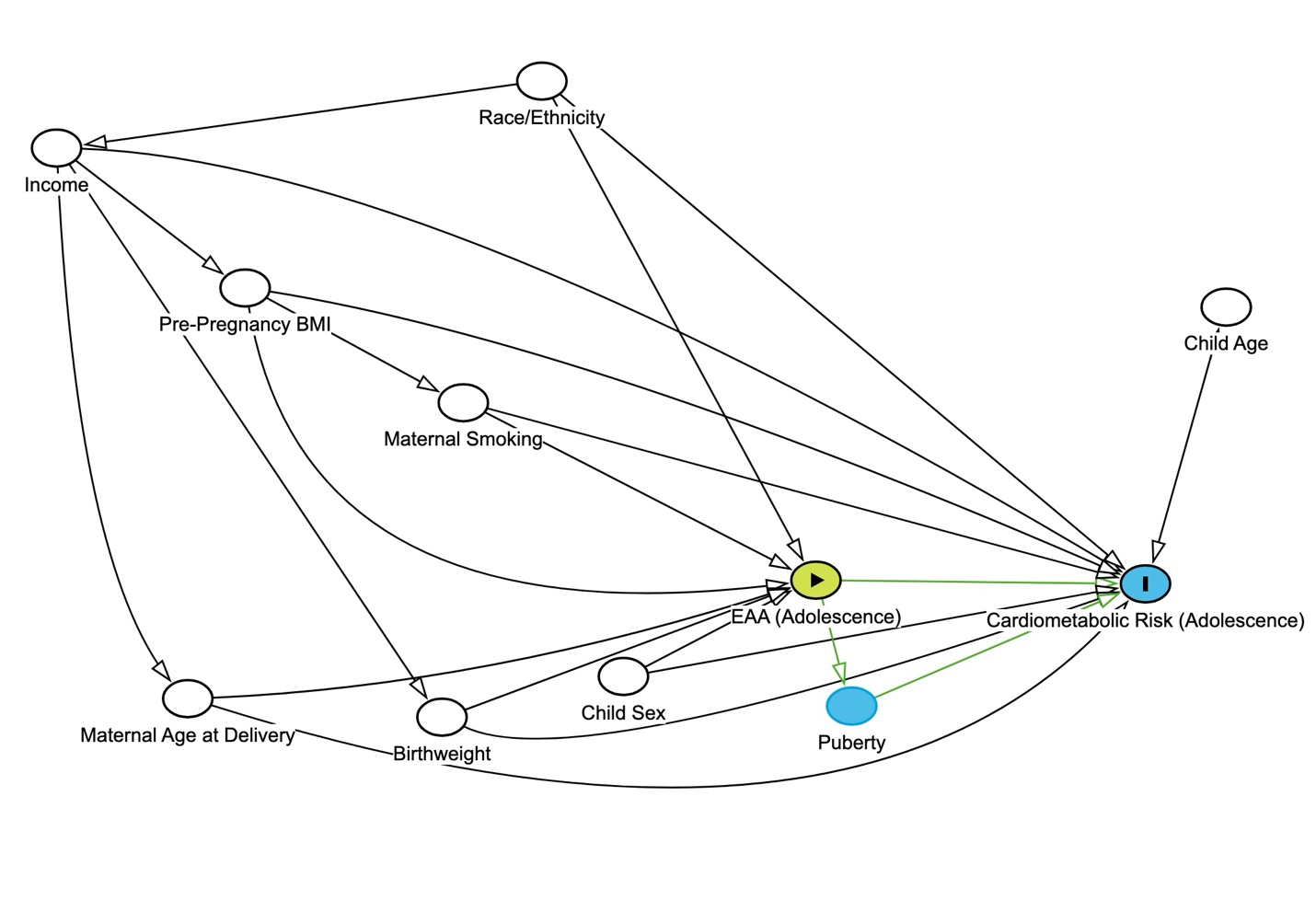


# Supplemental Figure 4. Directed acyclic graphs for the relationship of epigenetic age acceleration (EAA) at age 12 years with adolescent cardiometabolic risk.

Supplemental Table 1. Comparison of sociodemographic and perinatal characteristics of singleton children from the HOME Study included and excluded in the analyses of epigenetic gestational age acceleration (EGAA) at birth, and epigenetic age acceleration (EAA) and pace of biological aging at age 12 years with cardiometabolic risk.

|  | **Delivery** | |  | **12-Year** | |  |
| --- | --- | --- | --- | --- | --- | --- |
|  | **Included**,  N = 181 (45%)^a^ | **Excluded**,  N = 217 (55%)^a^ | **p-value**^b^ | **Included**,  N = 183 (46%)^a^ | **Excluded**, N = 215 (54%)^a^ | **p-value**^b^ |
| **Age^c,d^** |  |  | 0.103 |  |  | 0.077 |
| Median (IQR) | 39.4  (38.7, 40.0) | 39.0  (38.3, 40.0) |  | 12.3  (11.9, 12.8) | 12.5  (12.0, 13.1) |  |
| **Child Sex** |  |  | 0.589 |  |  | 0.903 |
| Female | 100 (55.2%) | 114 (52.5%) |  | 99 (54.1%) | 115 (53.5%) |  |
| Male | 81 (44.8%) | 103 (47.5%) |  | 84 (45.9%) | 100 (46.5%) |  |
| **Child Race** |  |  | 0.106 |  |  | 0.079 |
| Non-Hispanic White | 109 (60.2%) | 120 (55.3%) |  | 100 (54.6%) | 129 (60.0%) |  |
| Non-Hispanic Black | 62 (34.3%) | 72 (33.2%) |  | 71 (38.8%) | 63 (29.3%) |  |
| Other^e^ | 10 (5.5%) | 25 (11.5%) |  | 12 (6.6%) | 23 (10.7%) |  |
| **Maternal Age at Delivery (years)^d^** |  |  | 0.184 |  |  | 0.706 |
| 18 - 25 | 40 (22.1%) | 63 (30.3%) |  | 48 (26.2%) | 54 (26.2%) |  |
| > 25 - 35 | 116 (64.1%) | 113 (54.3%) |  | 109 (59.6%) | 121 (58.7%) |  |
| > 35 | 25 (13.8%) | 32 (15.4%) |  | 26 (14.2%) | 31 (15.0%) |  |
| **Annual Household Income** |  |  | 0.896 |  |  | 0.515 |
| < $20,000 | 38 (21.0%) | 50 (23.0%) |  | 46 (25.1%) | 42 (19.5%) |  |
| $20,000 - 40,000 | 30 (16.6%) | 37 (17.1%) |  | 30 (16.4%) | 37 (17.2%) |  |
| $40,000 - 80,000 | 63 (34.8%) | 77 (35.5%) |  | 59 (32.2%) | 81 (37.7%) |  |
| > $80,000 | 50 (27.6%) | 53 (24.4%) |  | 48 (26.2%) | 55 (25.6%) |  |
| **Pre-Pregnancy BMI (kg/m²)^d^** |  |  | 0.952 |  |  | 0.921 |
| < 25 | 76 (42.0%) | 89 (42.8%) |  | 76 (41.5%) | 89 (43.2%) |  |
| ≥ 25 - 30 | 59 (32.6%) | 69 (33.2%) |  | 62 (33.9%) | 66 (32.0%) |  |
| ≥ 30 | 46 (25.4%) | 50 (24.0%) |  | 45 (24.6%) | 51 (24.8%) |  |
| **Gestational Serum Cotinine (ng/mL)^d^** |  |  | 0.456 |  |  | 0.593 |
| <0.015 (Unexposed)^f^ | 55 (30.4%) | 61 (29.3%) |  | 52 (28.4%) | 64 (31.1%) |  |
| 0.015-3 (Secondhand) | 109 (60.2%) | 119 (57.2%) |  | 112 (61.2%) | 116 (56.3%) |  |
| >3 (Active Smoking) | 17 (9.4%) | 28 (13.5%) |  | 19 (10.4%) | 26 (12.6%) |  |
| ^a^ n (%) | | | | | | |
| ^b^ Wilcoxon rank sum test; Pearson's Chi-squared test; Fisher's exact test | | | | | | |
| ^c^ At delivery, age is reported as gestational age (weeks); at 12Y, it is reported as chronological age (years). | | | | | | |
| ^d^ Data on gestational age, maternal age at delivery, pre-pregnancy BMI, and gestational serum cotinine was not available for 9 singleton whose mothers dropped out prior to delivery, but re-enrolled at a later follow-up. | | | | | | |
| ^e^ Other category includes children of American Indian, Asian/Pacific, Hispanic, and Unknown race/ethnicity. | | | | | | |
| ^f^ Below detection limit. | | | | | | |


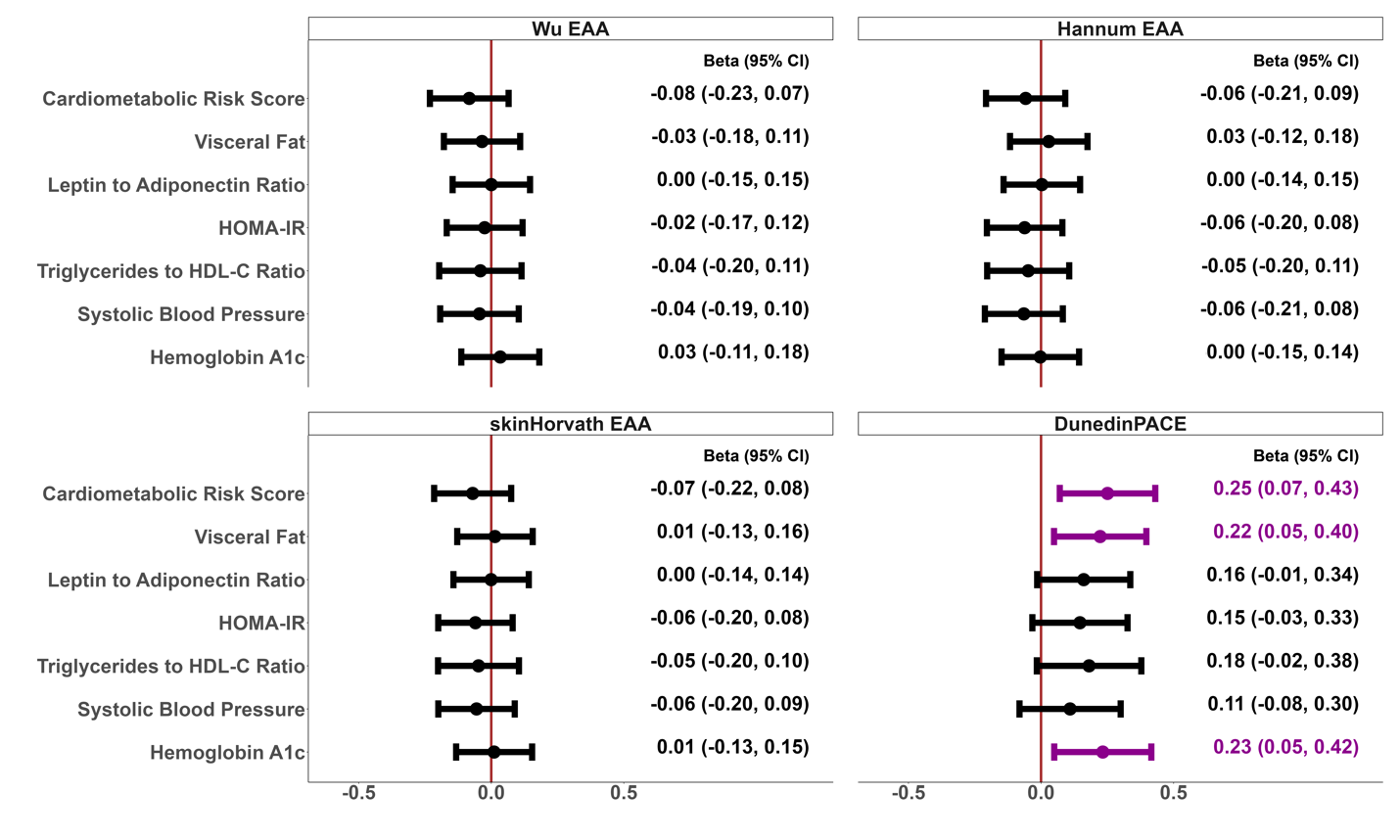


# Supplemental Figure 5. Forest plots of the adjusted associations of intrinsic epigenetic age acceleration (EAA) and pace of biological aging at age 12 years with adolescent cardiometabolic risk, adjusted for birthweight (sensitivity analyses).

Each circle represents the effect estimate of each association and the error bars represent the corresponding 95% confidence intervals. Associations with p<0.05 are displayed in purple.

Adjusted standardized difference in cardiometabolic risk score or individual cardiometabolic risk component at age 12 years per 1 SD increase in EAA/pace of biological aging at age 12 years. Intrinsic = adjusted for estimated cell type proportions CI = Confidence Interval; Adjusted for birthweight (continuous, grams) in addition to pre-pregnancy BMI (continuous, kg/m^2^), gestational serum cotinine (<0.015 ng/mL as unexposed; 0.015-3 ng/mL as secondhand exposure; >3 ng/mL as active smoking), maternal age at delivery (continuous, years), household income (continuous, USD/year), child age at outcome measurement (continuous, years), child sex (male; female), child race/ethnicity (non-Hispanic White; non-Hispanic Black; other), and estimated cell type proportions (at 12-year visit: neutrophils, eosinophils, basophils, monocytes, naïve and memory B cells, naïve and memory CD4 + and CD8 + T cells, natural killer, and T regulatory cells).


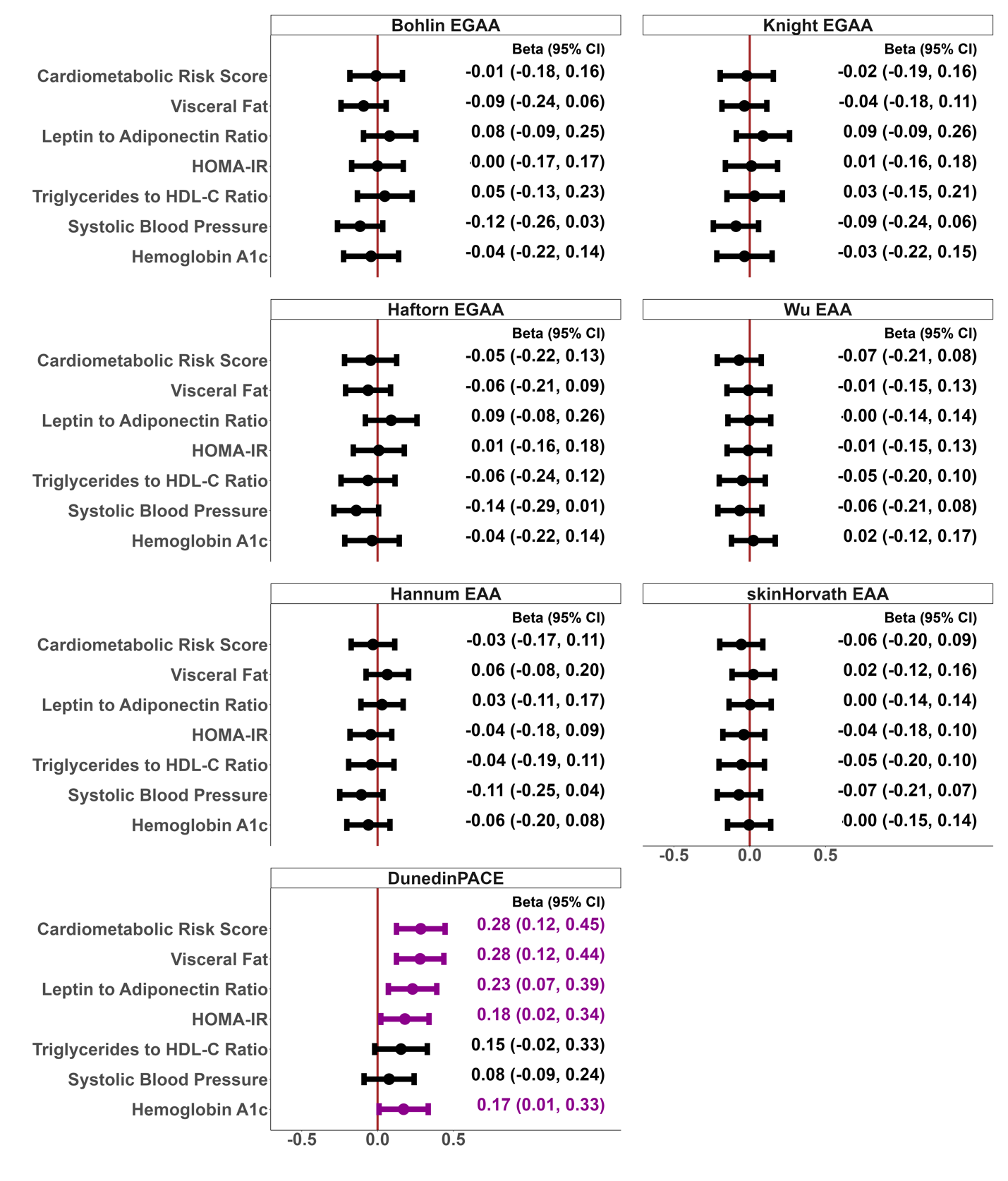


# Supplemental Figure 6. Forest plots of the adjusted associations of extrinsic epigenetic gestational age acceleration (EGAA) at birth, and epigenetic age acceleration (EAA) and pace of biological aging at age 12 years with adolescent cardiometabolic risk (sensitivity analyses): No adjustment for estimated cell type proportions.

Each circle represents the effect estimate of each association and the error bars represent the corresponding 95% confidence intervals. Associations with p<0.05 are displayed in purple.

Adjusted standardized difference in cardiometabolic risk score or individual cardiometabolic risk component at age 12 years per 1 SD increase in EGAA at birth or EAA/pace of biological aging at age 12 years. Extrinsic = not adjusted for estimated cell type proportions; CI = Confidence Interval; Adjusted for pre-pregnancy BMI (continuous, kg/m^2^), gestational serum cotinine (<0.015 ng/mL as unexposed; 0.015-3 ng/mL as secondhand exposure; >3 ng/mL as active smoking), maternal age at delivery (continuous, years), household income (continuous, USD/year), child age (continuous, years), child sex (male; female), and child race/ethnicity (non-Hispanic White; non-Hispanic Black; other). Associations of EAA/pace of biological aging with cardiometabolic risk at age 12 years were also adjusted for birthweight (continuous, grams).
